# Insurance data for research in companion animals: benefits and limitations

**DOI:** 10.1186/1751-0147-51-42

**Published:** 2009-10-29

**Authors:** Agneta Egenvall, Ane Nødtvedt, Johanna Penell, Lotta Gunnarsson, Brenda N Bonnett

**Affiliations:** 1 Department of Clinical Sciences, Faculty of Veterinary Medicine and Animal Science, Swedish University of Agricultural Sciences, SE-750 07 Uppsala, Sweden; 2 Department of Companion Animal Clinical Sciences, Norwegian School of Veterinary Science, N-0033 Oslo, Norway; 3 Agria Insurance, PO 70306, SE-107 23 Stockholm, Sweden; 4 Department of Population Medicine, Ontario Veterinary College, University of Guelph, Guelph, Ontario, N1G 2W1, Canada

## Abstract

The primary aim of this article is to review the use of animal health insurance data in the scientific literature, especially in regard to morbidity or mortality in companion animals and horses. Methods and results were compared among studies on similar health conditions from different nations and years. A further objective was to critically evaluate benefits and limitations of such databases, to suggest ways to maximize their utility and to discuss the future use of animal insurance data for research purposes. Examples of studies on morbidity, mortality and survival estimates in dogs and horses, as well as neoplasia in dogs, are discussed.

We conclude that insurance data can and should be used for research purposes in companion animals and horses. Insurance data have been successfully used, e.g. to quantify certain features that may have been hitherto assumed, but unmeasured. Validation of insurance databases is necessary if they are to be used in research. This must include the description of the insured population and an evaluation of the extent to which it represents the source population. Data content and accuracy must be determined over time, including the accuracy/consistency of diagnostic information. Readers must be cautioned as to limitations of the databases and, as always, critically appraise findings and synthesize information with other research. Similar findings from different study designs provide stronger evidence than a sole report. Insurance data can highlight common, expensive and severe conditions that may not be evident from teaching hospital case loads but may be significant burdens on the health of a population.

## Introduction

### Sources of information regarding animal populations

Background knowledge regarding the incidence and prevalence of disease in a population supports the diagnostic process and is needed for effective animal-health related services. Large-scale epidemiological studies are usually required for estimation of incidence and prevalence of diseases. Such studies can be conducted using primary or secondary data sources.

In primary data collection, data are assembled directly for the intended research purpose [1]. Only few large-scale population-based studies have been performed in companion animals. For example, data on all excised and laboratory-submitted tissues that could possibly be considered as cancers within a defined geographic area have been recorded [2-4]. In one of the studies the size of the base population was also estimated, allowing approximate determination of population-based rates of cancer [3]. In horses, disease information has been recorded together with information on the base-population of horses in 28 states of the US, enabling determination of population-based rates of broadly-defined disease problems [5,6].

Secondary data are those used for a purpose for which they were not primarily assembled [1]. Many different data sources can be considered secondary, such as retrospective evaluations of practice records from animal hospitals, breeding club registries and medical insurance data. Any registry that is used for research but where data collection was not specifically designed for the particular study should be considered secondary.

In descriptive observational small-animal or equine research, use of secondary data is very common. This is because primary data collection is expensive and time-consuming, especially when a large number of individuals are to be investigated. Because most practical observational study designs have limitations, determinants of disease will be most effectively mapped by comparisons among multiple studies with different designs. Extrapolation of findings to the general population, from even large-scale epidemiological studies in production animals or humans, must be done with caution. The use of secondary data in research is both justified and necessary. However, where secondary sources are used the data quality must be shown to be adequate in terms of accuracy (i.e. disease data are correct with respect to diagnosis) and completeness (i.e. an adequate amount of the entire disease load of the population is available). Of course, primary collection does not ensure accurate data, and it is possible for some secondary sources to be of very high validity, depending on how similar the research utilization is to the original purpose of the data collection.

### Existing registries

Registries have been frequently used to study different diseases in human epidemiology, especially within the Nordic countries (e.g. Denmark, Finland, Norway and Sweden), the UK and North-America. However, there are few well-designed disease registries for dogs, cats or horses that permit the calculation of incidence or prevalence of disease, survival rates or the evaluation of risk factors. To allow such calculations the registries need to have good documentation of the base population as well as of the occurring cases. The following are examples of existing registries that can be used as sources of secondary information regarding disease frequency and health in companion animal and horse populations.

#### Clinical records

Clinical records, either hard copy or electronic, from veterinary practices have been used extensively in companion animal research. Such medical records are likely to contain more complete documentation of clinical information (i.e. diagnoses) than clinical data subsequently entered into, for example, an insurance database. A limitation of hospital data is that the size and structure of the source population are unknown; therefore population-based estimates of incidence or prevalence are impossible. If the data are from a referral hospital, the diseased population might differ from the (unknown) catchment population in numerous ways as both clients and animals have passed through various filters, e.g. degree of diagnostic work-up and financial considerations, prior to arriving at the hospital. Among other possible biases, the patients might be selected towards more or less complicated cases depending on the kind of practice that is studied.

Few clinical databases are effectively monitored or validated. The Veterinary Medical Database Program (VMDP) registry was created in the 1960's [7,8]. Most of the North-American veterinary universities contribute data to this registry [9]. The VMDP amalgamates information from veterinary university animal hospitals but shares the limitations of individual-hospital data, being case-based. This large registry has been used to study general and specific diseases [7,10] as well as longevity [8]. Although various problems are inherent in these data, such as the mixture of referral and primary institutions and inconsistencies in data completeness or quality, this database has supported many studies that would not have been possible otherwise. If the disease studied is one which is likely to be referred to teaching hospitals, the data may reflect the occurrence of the problem within the general population (e.g. [11,12]).

#### Kennel club data - breeding registries

Kennel club, cat or horse breeding registries contain information about some portion of animals from a specific area, and data are usually recorded early in each individual's life. Screening program information may be completely incorporated into the database [13,14] if all results are entered. In other situations, results have been entered in an incomplete form [15] when it was the decision of the breeder/dog-owners whether or not to report the results. Clinical data or disease recordings are unlikely to be entered into such registries. Date of birth is probably fairly accurately recorded, but ancestry may be more rare [16]. The (proposed) completeness of date of birth recordings yields a large potential for longevity studies based on breeding registries, at least if combined with other information [17]. However, it has not, to our knowledge, been possible to register date of death in these ancestry databases with any reasonable degree of completeness or avoiding volunteer bias. However, given estimates of the longevity of the dogs from other sources, it is possible to approximate the current breed distribution and size of the population in a database with incomplete information on dates of death.

#### Insurance data

Databases from animal health insurance companies have become interesting to researchers because, unlike hospital data, they contain information on the background population as well as on clinical events. The insured population is followed from enrolment to termination of coverage. Medical insurance data have also been used in human medicine, but to a lesser degree than, e.g. "proper" disease registries from medical care. Animal insurance data have been used for research purposes since the 1970's [18,19] with increasing frequency during the last decade. Individual companion animals and horses can have veterinary care and/or life-insurance, however, insurance terms vary quite widely between companies and even more among countries and continents. In general, veterinary care insurance covers the costs of veterinary consultations/treatments and life-insurance reimburses the value of the animal in case of death.

### Objectives

The primary aim of this paper is to review the use of animal health insurance data in the scientific literature, especially in regard to morbidity or mortality in companion animals and horses. Methods and results were compared among studies on similar health conditions from different nations and years. The second objective was to critically evaluate benefits of and limitations for the use of insurance databases for research purposes. Finally, we aimed to discuss the future of research using animal insurance data and suggest steps to take to maximize their utility.

## Methods

For the purposes of this review, directed searches of PubMed and Web of Knowledge were made using appropriate terms, e.g. insurance and animal. An attempt has also been made to access ancillary literature, e.g. theses as well as refereed publications. The authors have been following the literature on morbidity and mortality in companion animals for the past 15 years, and major studies within the field are unlikely to have been missed in this search-process. The relevant literature was deemed insufficient in scope and content to allow for a formal systematic review, therefore a general and critical description has been produced. It should be noted that the majority of literature in this field has been produced by these authors using the database of one large Swedish insurance company (Agria Djurförsäkring).

## Results- Usage of insurance data in research

### Publications based on animal insurance data

Papers based on animal insurance data in the scientific literature are shown in table 1 for dogs (n = 19) and table 2 for horses (n = 13). The focus is on refereed publications. Therefore, although theses and proceedings (if later followed by published articles) are reviewed, they are not included in the tables. Taken together with some posters, conference presentations and theses, no other publications concerning dogs, cats and equines were found where insurance data had been used. Some German theses on equine disease have been excluded (see [20,21]). The tables provide a brief summary of the country, time-period, whether data on mortality or morbidity are presented and the disease problem(s) (general or specific). As can be seen from the two tables, most of the published studies using animal insurance data are from the Agria insurance company in Sweden and by the authors of this review. The following sections contain some examples of use of insurance data in research, with a comparison of results from different populations where feasible.

**Table 1:** Published studies (n = 19) based on data from insured dogs or using insured dogs as a sampling frame in chronological order

**Source**	**Year**	**Country**^**1**^	**Main focus/disease**^**2**^
Bergsten *et al*. (1978) [18]	1975-1977	Sweden	Morbidity and mortality; general and by cause
Häggström *et al*. (1992) [55]	1980-1990	Sweden	Mortality and morbidity; myxomatous valve disease
Bonnett *et al*. (1997) [22]	1992, 1993	Sweden	Mortality; general and by cause
Egenvall *et al*. (2000) [23]	1995, 1996	Sweden	Morbidity and mortality; general
Egenvall *et al*. (2000) [24]	1996	Sweden	Morbidity; general, by cause
Egenvall *et al*. (2000c) [36]	1995, 1996	Sweden	Mortality; general
Egenvall *et al*. (2001) [56]	1995, 1996	Sweden	Morbidity; pyometra
Dobson *et al*. (2002) [31]	1997-1998	UK	Morbidity; neoplasia
Edwards *et al*. (2003) [32]	1997-1998	UK	Morbidity: lymphoma
Bonnett *et al*. (2005) [33]	1995-2000	Sweden	Mortality; general, by cause
Davison *et al*. (2005) [57]	Not stated	UK	Diabetes mellitus^3^
Egenvall *et al*. (2005) [58]	1995-2000	Sweden	Mortality; general, by cause
Egenvall *et al*. (2005) [34]	1995-2002	Sweden	Morbidity and mortality; mammary tumours
Bergström *et al*. (2006) [47]	1995-2002	Sweden	Morbidity; Caesarean section
Egenvall *et al*. (2006) [59]	1995-2002	Sweden	Mortality; heart disease
Nødtvedt *et al*. (2006) [60]	1995-2002	Sweden	Morbidity; atopic dermatitis
Nødtvedt *et al*. (2007) [43]	1995-2002	Sweden	Morbidity; atopic dermatitis, spatial distribution
Egenvall *et al*. (2007) [35]	1995-2002	Sweden	Morbidity and mortality; osteosarcoma
Fall *et al*. (2007) [61]	1995-2004	Sweden	Morbidity and mortality; diabetes mellitus

^1 ^Data from Swedish studies (n = 16) emanates from Agria insurance  and data from UK (n = 3) from PetProtect

^2 ^All included studies have included incidence measures

^3 ^morbidity/mortality not stated

**Table 2:** Published studies (n = 13) based on data from insured horses in chronological order

**Source**	**Year**	**Country**^**1**^	**Main focus/disease**
Greenhall *et al*. (1979) [19]	1978-1979	US^2^	Mortality; by cause^3^
Bergsten *et al*. (1983) [62]	1973-1981	Sweden	Mortality; locomotor problem
Clausen *et al*. (1990) [29]	1977-1987	Germany^4^	Morbidity; general. by cause^2^
Hommerich *et al*. (1995) [20]	1984-1994	Germany^5^	Mortality; by cause
LeBlond *et al*. (2000) [28]	1995	France^6^	Mortality; general, by cause
Egenvall *et al*. (2005) [25]	1997-2000	Sweden	Morbidity; general
Penell *et al*. (2005) [26]	1997-2000	Sweden	Morbidity; by cause
Egenvall *et al*. (2006a) [27]	1997-2000	Sweden	Mortality; general, by cause
Egenvall *et al*. (2006b) [63]	1997-2002	Sweden	Mortality; general, locomotor problems
Higuchi (2006) [51]	2001-2003	Japan	Morbidity and mortality; colic
Egenvall *et al*. (2008) [39]	1997-2002	Sweden	Morbidity; general, locomotor problems
Egenvall *et al*. (2008) [40]	1997-2002	Sweden	Morbidity and mortality; colic
Egenvall *et al*. (2009) [41]	1997-2002	Sweden	Morbidity and mortality; riding schools horses, locomotor problems

^1 ^Data from Swedish studies (n = 8) emanates from Agria insurance  and various companies provided data for the other studies

^2 ^Rhulen Agency, Inc, Monticello NY

^3 ^only proportional measures presented

^4 ^not stated

^5 ^Vereinigten Tierversicherung Gesellschaft a.G.

^6 ^data from 9 of 42 identified French horse insurance companies.

#### General morbidity and mortality

In general, insurance data will be a source of information on the disease load of the insured animals. This holds true as long as disease events have veterinary care costs attached to them, that animal owners claim those to the insurance company and that these events are covered by the insurance. Swedish insurance data have been used to study general mortality and morbidity in dogs and horses, both with respect to incidence and proportional measures [22-27]. In general, only disease events for which the cost exceeds the deductible will be recorded. Different levels and applications of the deductible will influence comparability across data sources (see further information below). Distinguishing between death and euthanasia is not possible in the Agria data. In this insurance program most medical and traumatic problems were covered, whereas, e.g. behavioral issues or non-traumatic tooth-problems were not. The number of exclusions from coverage varies somewhat by breed and has been tending to increase over time (personal communication, Lotta Gunnarsson, Agria Insurance) but, during the period of these studies, exclusions and limitations were relatively few. Some insurance programs/companies have highly restrictive or individualized coverage, and researchers cannot be certain that all disease events will be recorded equally for all insured animals. For Swedish insurance companies, clients are not "punished" with higher insurance fee or exclusion from insurance if they use the insurance which reduces the risk of not reporting disease problems in the animals.

The published mortality from the Swedish insurance database could be defined in two ways, either as total mortality including all registered deaths or as diagnostic mortality, i.e. when an insurance claim (with a cause of death/reason for euthanasia/diagnosis) was submitted. The annual total and diagnostic mortality of dogs were 3 and 2%, respectively, and for horses both 4% [22,23,27]. These estimates varied with breed, gender and age in both species and also with geographic location in horses [22,23,27]. Among insured horses in France, an overall mortality rate of 2.47% was reported and the most common cause of death was death as a consequence of foaling (dead colts were also relatively common) followed by colic and locomotor disease [28]. From the Swedish insurance database, such foaling complications, nor dead colts, were common reasons for death - although the condition is covered by the complete insurance form (as well as by other types of insurance). However, locomotor problems have repeatedly been found to be the most common cause both for morbidity and mortality in insured horses [20,26,27,29].

From the Agria insurance database, the most common specific causes of death in dogs are tumour, trauma, locomotor and heart problems [22]. In dogs, the most common causes of morbidity were skin, digestive, genital and respiratory tract problems [24]. In horses, the most common reasons for mortality were; joint, skeletal, hoof and digestive disorders and for veterinary care events; joint, skin, digestive and skeletal disorders [26,27]. However, behavioral problems have been shown to be a common cause of canine euthanasia in Denmark [30]. Because such problems are generally not covered by the Agria insurance, any discrepancy between total and diagnostic mortality may be (at least partially) accounted for by unclaimed behavior problems. This may vary across breeds. For example, for mixed breed dogs the proportion of all deaths that were claimed was around 50%. In some breeds, e.g. Bernese Mountain Dogs and Cavalier King Charles Spaniels, over 80% of deaths have an associated diagnosis and behavior problems are unlikely to account for many deaths. Other reasons may influence the lack of claims, however, including owner reluctance to receive money following the death of their pet or simple oversight.

Even though there are among-study similarities, it is expected that comparisons between breeds or across ages are best done within each study. This is because of underlying differences in, e.g. insurance policies, analytical methods or time effects.

#### Neoplasia

Dobson and co-workers [31] published rates of canine neoplasia based on data from an insurance company in the UK, where case records were scrutinised for classification of tumour type. The rates were age-standardised to an estimated composition of the UK dog population. Statistics on lymphoma in dogs were published from the same material [32]. From the Swedish insurance database [33-35], rates have been constructed for the incidence of mammary tumours (age-standardised from UK 205 per dogs per 100,000 dogs/year and crude from Sweden 1110 dogs per 100,000 dog-years at risk) and osteosarcoma (from UK: 57 per dogs per 100 000 dogs/year (age-standardised); from Sweden 55 dogs per 100,000 dog-years at risk), as well as for the overall neoplasia rate (from UK crude and standardised 1948 and 2671 cases per 100 000 dogs/year and crude mortality from Sweden 500 deaths per 100,000 dog-years at risk) the latter reflected solely from life-insurance claims. Crude and age-standardised rates of lymphosarcoma of 79 and 107 cases per 100 000 dogs/year, respectively, was estimated for dogs in the UK, and from Sweden a crude mortality (life-insurance claims) of 90 deaths per 100,000 dog-years at risk [31-33]. In conclusion, the rates of osteosarcoma and lymphosarcoma are similar between the two countries, while discrepancies are larger for the other diseases. The likely reason for this similarity may be that the former diseases are highly malignant and therefore most owners will seek medical care and the disease occurrence will be registered if the dog has insurance (the owner will seek medical advice because the dog has moderate to severe clinical signs and the condition likely becomes diagnosed because it is relatively simple to determine the diagnosis). When the conditions are less malignant, insurance-, owner- and dog-related factors, as well as prognostic and cost considerations about pursuit of, e.g. diagnostic work-up or therapy, may all influence access to veterinary care and subsequent entry of information to the database.

#### Survival estimates

Survival estimates for dogs up to 10 years of age and horses up 22 years of age have been presented based on Swedish animal insurance data [27,36]. These rates agree well with those from primary data collection [37], and with survival estimates from horses entering quality contests [17]. However, estimated canine survival from the VMDP database [8] agreed poorly with the results from Sweden, which we believe mainly is caused by the fact that the VMDP database solely contains cases. The conclusion is that estimates of length of life should be possible to derive from life-insurance databases with good coverage. Because life-insurance coverage is likely to terminate at a certain age, these calculations will only be possible to that age. Furthermore, all insured animals will have already survived to the age at which they were insured and estimates from insurance data disregard deaths occurring at very young age.

### Frame for gathering study populations, adding extra data from interviews or practice records

In England, an insurance database was used as a sampling frame for interviews about causes of canine death [38]. The possibility for identifying cases and high risk groups from insurance databases can support various research designs, with due consideration of confidentiality issues (see below).

### Costs of veterinary care

It is inherent in insurance veterinary care data that veterinary care costs are attached to each receipt. A few times it has been possible to demonstrate the gross cost of general or specific veterinary care [39-42]. For example, a substantial increase in costs for general veterinary care in horses was found over an 8-year time period using Swedish data. Between the years 1997 and 2004, the increase in costs per claimed horse was 59% and the increase in cost per horse-year at risk was 41%, compared to a consumer's price index increase of only 10% [42]. Veterinary costs are an increasingly important factor in veterinarian-client-animal interactions and, in spite of limitations relative to the nature of insurance reimbursements the information is useful. Findings from longitudinal studies of subsequent risk following initial diagnosis of disease problems from insurance data could ideally be combined with clinical outcome evaluations in an effort to determine effective management strategies and to support diagnostic decision making.

## Benefits of and limitations for using animal insurance data in research

### Benefits of using animal insurance data in research

Obvious benefits of insurance databases are that they already exist and contain information that can be accessed. Given that an insurance company gives permission to access data, they become relatively straightforward to manage, even if many computational problems may arise during the process. Still, there is less work and lower cost compared to assembling a similar amount of information through primary data collection. Primary data collection, of course, is not free of limitations, e.g. representativeness of the sample for the target population, volunteer bias and non-response issues.

Many insurance databases are large and high statistical power can be achieved. They are therefore relatively well suited to analyse for example breed effects. Dog breeds differ widely in size and conformation as well as regarding disease patterns. Unfortunately, in most observational studies interactions between dog breeds and other factors are rarely explored simply because there are often few dogs of many different breeds.

An insurance database contains detailed information about the addresses of the owners. Because of billing and communication reasons the locations of the owners are regularly updated. Our group studied the impact of geographic factors (e.g. distribution of specialised veterinarians) on the occurrence of atopic dermatitis among dogs insured by Agria using spatial analysis [43]. However, it may be more difficult or impossible to follow the movement of persons and animals over time and the assumption that the animal resides solely at the location of the owner's primary address may not always be valid and is likely less accurate for horses than dogs.

Our experience is that data can be handled so as to maintain confidentiality of client and animal information. It is generally not an objective to describe individual animals, therefore confidentiality can be maintained. However, the ethical and legal considerations of the data usage must always be considered, especially if there is any linkage to other databases or information that could be used to identify individuals.

### Limitations of using animal insurance data in research

#### Validity of the data

Once compiled, a "research insurance database" is technically easy to analyse for a number of disease problems. However, different problems may require various strategies and precautions. Most of the Swedish publications have analysed problems at a rather crude level of diagnostic detail, where the authors have been satisfied with the data validity. The major challenge for more specific diagnoses is to correctly identify (all) the cases of interest. This is influenced not only by the data, but by e.g. the acumen of the veterinarians supplying the data and even the general culture of access to care relative to specific conditions. Factors specific to each disease/problem proposed for study must be considered, optimal strategies employed, or, in the case of, e.g. unavailable or inaccurate data, the study of that condition abandoned. Some "simple" diagnoses might have one easily identifiable code, be correctly coded to a large extent (have a high positive predictive value) and be found in the database when present (have a high observed sensitivity). However, for many complex disease problems these conditions may not be satisfied. Review of original practice records, validation of specific diagnoses or other more intensive strategies may be needed to supplement the insurance data.

In the Swedish insurance database used in research, all veterinarians provide diagnoses using a standardized diagnostic registry [44]. This provides a level of consistency but the underlying accuracy is unknown. Veterinarians often use non-specific codes (e.g. 'dead, no diagnosis, or 'clinical sign of illness'). This may reflect the realities of veterinary practice, but is, of course, a limitation to detailed investigation. Often, only one diagnostic code is allowed for each receipt. Validation has shown this to result in high correctness, i.e. the animal did experience that event [45,46]. However, such a system also reduces completeness, as not all the problems an animal experiences will be recorded.

For some conditions, e.g. Caesarean section in bitches [47] coding is likely very accurate. However, because of insurance company restrictions, it was only possible to study the first event of Caesarean section. As another example of a specific condition, a sample of records from dogs with the diagnosis atopic dermatitis was investigated [48]. Of the scrutinized cases, all were recorded by the submitting veterinarian to be suffering from canine atopic dermatitis and 98% were judged by the principal investigator as having allergic skin disease. However, for a large number of dogs cutaneous adverse food reactions had not been properly ruled out and in total it was suggested that approximately 75% of the cases had canine atopic dermatitis or canine atopic dermatitis with concurrent cutaneous adverse food reactions.

Continuous monitoring and validation of secondary databases is challenging and generally not followed to an adequate extent in those limited sources reported for companion animals. The Agria insurance database was initially validated for dogs and for horses [45,46] showing that diagnostic agreement approached 85%, while for demographic variables it was > 94% [45,46]. However, monitoring the health situation over time can be a challenge if insurance terms (e.g. premiums, relative deductibles and maximal reimbursements) change significantly.

#### External validity (Representativeness of the population)

The extent to which findings from an insured population can be extrapolated to other populations is difficult to assess. The proportion of a population in a country that is insured by a given insurance company should be accounted for. For example in Sweden, around a third of all horses have complete insurance by Agria (excluding all racing horses) and the same share of the dog population is estimated to be covered by the same company (a majority of all dogs are insured). Insurance coverage has been shown to vary somewhat by breed [49] and if insurance coverage ends at a certain age, old dogs will not be covered and statistics will not be relevant for these. It has also been determined that this insured population was reasonably representative of the general Swedish dog population with regard to feeding and exercise [50].

Findings for morbidity and mortality for insured animals cannot be presumed to apply to the uninsured population. Veterinary care may be accessed less by owners of non-insured compared to insured animals. Uninsured animals may also have fewer medical procedures performed. Even mortality, based on decisions to euthanize animals, may be different depending on insurance status.

Also insurance enrolment may vary related to the use/function of the animal. If so, the extent of disease and injury, if associated with usage, may vary from the insured to the non-insured population. This is likely true, but, for the Swedish population, it may not be as significant as one might suppose, even for the horse population, as both high-level competition (non-racing) horses and backyard horses are insured to a large extent (personal observations). However, the likelihood of the owner accessing veterinary care seems to vary even within the insured population, perhaps influenced by personal, geographical and economic factors (unpublished information).

### Benefits of and limitations for comparison across studies

Notwithstanding all the limitations which must be duly considered, it is possible and useful to compare appropriately compiled research findings within the insured population of an individual company, across companies, within a country or across countries. In Sweden, we believe that we can extrapolate to the total insured-dog population in this country. In addition, for many specific breeds we believe the findings are informative outside of Sweden. Many dog breeds in Sweden have close genetic links to breeding lines in UK and other European countries. Where diseases may be a function of, for example, size or function, extrapolation to similar dogs in other countries is reasonable. For horses there is a limitation that actively competing/training trotters and thoroughbreds have "less" coverage, mainly because the insurance forms for such horses do not cover all types of medical problems. With this exception, findings from the Agria horse population are likely informative both within and outside of Sweden. Extrapolation should be done cautiously, with due consideration of possible similarities to or differences from the Swedish populations and situation. However, studies on the same disease complexes from different countries are welcomed for comparison. Smaller differences in results between national populations might very well be due to different strategies of insuring animals (for example the ease and degrees that people can switch company, or reinsure an animal that has quit insurance, or policies of continued coverage when owners have used the insurance for expensive veterinary care).

It can be seen from the above that several issues can complicate between-study comparisons, best exemplified from the neoplasia section. Even if the same disease is studied, different incidences may be estimated if studying all claims (i.e. veterinary care) compared to only life-insurance claims (deaths). We believe one of the major reasons for the observed differences when comparing results between insurance databases or between different sources is the possibility for inclusions of different type of cases. For example, colic cases from an insurance database are likely in general more severe [40,51] compared to those from a primary study with data from animal holders [5,52], even though cases will be less severe than in a study on surgical colic from a hospital database [53]. (I.e. the cases in the insured population will not be found in the database until they have reached the deductible, animal holders will register all colics including self-limiting problems and surgical colic cases are by definition not eligible until costly surgery has taken place.) Many issues that complicate comparison across studies are not unique to insurance data, and must always be kept in mind when extrapolating information from the literature.

Judging by the number of publications, it is clear that the Swedish insurance company Agria has been extremely open about sharing their data with the scientific community, perhaps more so than other companies world-wide. This has allowed for a thorough description of at least this insured population. For example, censoring (withdrawal) rates have been reported from many of the Swedish studies, whereas the dynamics of the other databases have seldom been demonstrated. Comparing Swedish horses and dogs, the censoring rates of the horses are much higher. As horses are sold and bought to a much larger extent this feature is expected, but we believe it is an advantage to be able to document it as well.

## Conclusion and future use of insurance databases

Our research-group intends to continue analysis of Swedish animal insurance statistics for dogs and horses and to also include cats in the future. Perhaps use of animal insurance data from Sweden and the UK will expand and companies in other countries will allow researchers to access their data. However, detailed information on the insurance policies, populations and data must be available to address the concerns and limitations described above. The basic tenet of animals being 'at risk' must be satisfied. In other words, there must be realistic assurance that if an insured animal experienced an event of interest, it would be recorded within the database. It is quite likely that given changes in the economy, in general, and with increasing costs of veterinary care, there may be changes in the animal insurance industry. Increased restrictions, limitations on coverage and increased individualization of policies may impact the usefulness of insurance data for research.

A possible extension of research based on insurance data is the construction and dissemination of detailed statistics not only back to the insurance companies but especially to breed clubs, breeders and owners. The latter has recently been launched using data from Agria, where statistics have been compiled and distributed to Swedish breed clubs (for breeds with a reasonably large base population) [54]. Recent focus on diseases in purebred dogs underscores the importance of getting health information into the hands of the end-users, i.e. dog breeders, in a user-friendly format with pertinent content.

We conclude that insurance data can and should be used for research purposes in companion animals and horses. They are simply too useful of a resource to ignore as they can fill certain gaps left by other types of research. Insurance data have been successfully used, e.g. to quantify certain features that may have been hitherto assumed, but unmeasured. Validation of insurance databases is necessary if they are to be used in research. This must include the description of the insured population and an evaluation of the extent to which it represents the source population. Data content and accuracy must be determined over time, including the accuracy/consistency of diagnostic information. Readers must be cautioned as to limitations of the databases and, as always, critically appraise findings and synthesize information with other research. Similar findings from different study designs provide stronger evidence than a sole report. Insurance data can highlight common, expensive and severe conditions that may not be evident from teaching hospital case loads but may be significant burdens on the health of a population.

## Competing interests

The authors declare that they have no competing interests. BNB is presently an independent, part-time consultant to the Agria Insurance company.

## Authors' contributions

AE drafted the manuscript and compiled the insurance literature. All authors made substantial input to the review, critically discussed the progressing manuscript and approved the final manuscript.
